# MiR‐221‐3p Attenuates IL‐33‐Induced Mast Cell Cytokine Expression by Targeting KIT

**DOI:** 10.1002/alr.23558

**Published:** 2025-03-25

**Authors:** Ruowu Liu, Jiao Zhou, Jing Zhou, Feng Liu, Yafeng Liu, Juan Meng, Luo Ba, Hengyi Xiao, Shixi Liu, Nan Zhang, Claus Bachert, Jintao Du

**Affiliations:** ^1^ Department of Otolaryngology‐Head and Neck Surgery West China Hospital, Sichuan University Chengdu China; ^2^ Department of Medicine and Engineering Interdisciplinary Research Laboratory of Nursing & Materials West China Hospital, Sichuan University Chengdu China; ^3^ Department of Otolaryngology People's Hospital of Tibet Autonomous Region Lhasa China; ^4^ Department of Aging and Geriatric Medicine, National Clinical Research Center for Geriatrics, State Key Laboratory of Biotherapy West China Hospital, Sichuan University Chengdu China; ^5^ Department of Otorhinolaryngology‐Head and Neck Surgery University Hospital of Münster Münster Germany

**Keywords:** CRSwNP, IL‐33, KIT, mast cell, miR‐221‐3p

## Abstract

**Background:**

Mast cells (MCs) are involved in type 2 inflammation in chronic rhinosinusitis with nasal polyps (CRSwNP), which depends on interleukin (IL)‐33 stimulation. MiR‐221 is reported to be an important regulator of MCs, and miR‐221‐3p can be expressed in CRSwNP. However, the role of miR‐221‐3p in CRSwNP is unclear.

**Methods:**

Ethmoid tissues from control subjects (*n* = 12) and polyps from patients with CRSwNP (*n* = 40) were collected. The expression of miR‐221‐3p and cytokines was detected by real‐time quantitative polymerase chain reaction (qPCR). The activation of P65 and ERK was determined by western blotting. The localization of miR‐221‐3p was detected via in situ hybridization combined with immunofluorescence (IF), and its target was identified via a luciferase reporter system. Human MCs were incubated with IL‐33 or stem cell factor. MicroRNA mimics/inhibitor and lentiviral plasmids were used to determine the role of miR‐221‐3p in MCs.

**Results:**

We observed increased expression of miR‐221‐3p in CRSwNP, and localized its expression in MCs. The expression of miR‐221‐3p was negatively correlated with that of IL‐4, IL‐5, and IL‐13 in CRSwNP. MiR‐221‐3p can be induced by IL‐33 in MCs and plays a negative regulatory role in cytokine expression and signaling pathways in IL‐33‐induced MC activation. As the direct target of miR‐221‐3p, the receptor KIT was negatively correlated with miR‐221‐3p and decreased in CRSwNP. In MCs, KIT is essential for an effective response to IL‐33 stimulation. We here demonstrated that miR‐221‐3p regulates cytokine expression by targeting KIT in IL‐33‐activated MCs.

**Conclusions:**

MiR‐221‐3p inhibits MC‐dependent type 2 inflammatory conditions, rendering it a negative regulator of CRSwNP.

**Abbreviations**
CRSwNP: chronic rhinosinusitis with nasal polyps
ECRSwNP/EOS‐NP: eosinophilic chronic rhinosinusitis with nasal polyps
FISH: fluorescence in situ hybridization
HPF: high‐powered field
IF: immunofluorescence
IL: interleukin
ILC2s: group 2 innate lymphoid cells
miR/miRNA: microRNA
nECRSwNP/nEOS‐NP: noneosinophilic chronic rhinosinusitis with nasal polyps
NPs: nasal polyps
qPCR: real‐time quantitative PCR
RT: room temperature
SCF: stem cell factor
Th2: T‐helper 2 cells
WB: western blotting

## 1 Introduction

Chronic rhinosinusitis with nasal polyps (CRSwNP) is a heterogeneous disease that has historically been divided into two subtypes on the basis of the degree of infiltrated eosinophilia in nasal polyps (NPs): noneosinophilic CRSwNP and eosinophilic CRSwNP [1]. Compared with noneosinophilic CRSwNP, eosinophilic CRSwNP presents challenging clinical problems characterized by severe nasal symptoms, high recurrence rates, and comorbidities. Eosinophilic CRSwNP is the most common CRSwNP in Western countries, and it has also become more common in Asian countries in recent decades [2]. Eosinophilic CRSwNP is characterized by type 2 inflammation, characterized by elevated interleukin (IL)‐4, IL‐5, and IL‐13 concentrations and tissue infiltration of eosinophils. Studies have suggested that type 2 inflammation in CRSwNP is controlled mainly by T‐helper 2 (Th2) cells and group 2 innate lymphoid cells (ILC2s), and the importance of mast cells in this process has been emphasized by recent studies [1, 3].

Mast cells are known for their pivotal role in allergic reactions, which can release allergic mediators through the pathway of antigen‐specific activation [4]; the discovery of the IL‐33 receptor ST2 on mast cells revealed that mast cells also play a critical role as amplifiers of IL‐33‐mediated type 2 inflammation [5]. After binding to ST2 on mast cells, IL‐33 can activate the ERK1/2 signaling pathway to promote IL‐5 and IL‐13 transcription as well as the NF‐κB signaling pathway to promote tumor necrosis factor (TNF) transcription [6, 7]. In asthma, IL‐33 stimulates type 2 gene expression in mast cells, resulting in sustained type 2 inflammation, which is associated with severe asthma [8, 9]. Studies have shown that the number of mast cells is increased in eosinophilic CRSwNP and that activated mast cells are positively correlated with type 2 cytokines and eosinophils, indicating that mast cells are closely associated with type 2 inflammation in CRSwNP [10, 11]. IL‐33 can be released by nasal epithelial cells and triggers type 2 inflammation in eosinophilic CRSwNP via the activation of Th2 cells and ILC2s [12, 13]. The role of mast cells, which are potent effector cells via IL‐33, in CRSwNP needs further study.

MicroRNAs (miRNAs) are a class of small noncoding RNAs with a length of approximately 22 nucleotides that regulate gene expression after transcription and participate in a variety of pathological processes [14]. Many differentially expressed miRNAs, some of which can regulate inflammatory responses and play important roles in the pathogenesis of CRSwNP, have been detected in CRSwNP [15]. A recent study analyzing the miRNA expression profile of CRSwNP from China revealed that the expression of miR‐221‐3p was greater in the CRSwNP group than in the control group [16]. Interestingly, another study from Brazil analyzing the miRNA expression profile also showed the same results [17]. We therefore hypothesized that miR‐221‐3p might affect the pathogenesis of CRSwNP.

In the present study, we identified the expression and cell location of miR‐221‐3p and the correlations between miR‐221‐3p and cytokines in CRSwNP. Next, we examined the role of miR‐221‐3p in regulating cytokine expression in IL‐33‐activated mast cells. Furthermore, we validated the expression of KIT, a target gene of miR‐221‐3p, in CRSwNP and its role in the expression of cytokines in activated mast cells.

## 2 Methods

### 2.1 Patients and Samples

Control subjects and patients with CRSwNP were recruited into the study. The diagnostic criteria for CRSwNP were based on the European Position Paper on Rhinosinusitis and Nasal Polyps 2020 [18]. The atopic status of the patient was confirmed by the skin prick test, and the patient's comorbidities and basic demographic data were documented preoperatively. Normal ethmoid tissue was obtained from control subjects during septoplasty. NPs were collected from patients with CRSwNP during functional endoscopic sinus surgery. All the samples were divided into three parts and processed as described previously [19]. The characteristics of the control subjects and patients with CRSwNP are shown in Table S1. This study was approved by the Medical Ethics Committee of the West China Hospital of Sichuan University (No. WCH2015‐199) and conducted in accordance with institutional guidelines. Written informed consent was obtained from all patients prior to the study.

### 2.2 Histological Analysis

The samples were embedded in paraffin wax and subsequently sectioned at a thickness of 5 µm. The sections from both control subjects and patients with CRSwNP were stained with hematoxylin and eosin to assess the extent of eosinophil infiltration. CRSwNP can be further classified into two subtypes: eosinophilic CRSwNP (EOS‐NP) and noneosinophilic CRSwNP (nEOS‐NP). EOS‐NP was defined as meeting the criterion of having more than 10 eosinophils per high‐powered field (HPF) in three randomly selected fields, whereas those failing to meet this standard were classified as nEOS‐NP [18].

### 2.3 Isolation of Total RNA and Real‐Time Quantitative PCR

The total RNA from the samples and cells was isolated via the Animal Total RNA Isolation Kit (FOREGENE, China) and the Cell Total RNA Isolation Kit (FOREGEN), respectively. MiRNAs were reverse transcribed into cDNAs via the All‐in‐One™ miRNA First‐Strand cDNA Synthesis Kit (GeneCopoeia, China), whereas mRNAs were reverse transcribed into cDNAs via the HiScript III All‐in‐one RT SuperMix Perfect for real‐time quantitative polymerase chain reaction (qPCR) (Vazyme Biotech, China). The qPCR of miRNAs was conducted with the All‐in‐One™ miRNA qRT‐PCR Detection Kit (GeneCopoeia), and for mRNAs, Taq Pro Universal SYBR qPCR Master Mix (Vazyme Biotech) was utilized. Relative expression levels were normalized to those of U6 and GAPDH for miRNAs or mRNAs, respectively. The sequences of primers used in this study are detailed in Table S2.

### 2.4 Fluorescence In Situ Hybridization

Fluorescence in situ hybridization (FISH) was performed as described previously [19]. In brief, 8 µm‐thick cryosections were prepared from frozen samples embedded in O.C.T. compound. Subsequently, FISH was carried out via the Enhanced Sensitive in situ hybridization (ISH) Detection Kit V (FITC; Boster, China) following the manufacturer's instructions. Locked nucleic acid (LNA) probes labeled with 5′‐digoxigenin (DIG) and 3′‐DIG were utilized for ISH. The detection probes for miR‐221‐3p, along with the positive control (U6) probe and negative control (scramble‐miR) probe, were synthesized by Takara (Takara, China).

### 2.5 Immunofluorescence

Paraffin sections from both control subjects and patients with CRSwNP were prepared by deparaffinization, hydration, and antigen retrieval. The sections were then permeabilized with phosphate‐buffered saline (PBS) containing 0.1% Triton X‐100 at room temperature (RT) for 15 min and subsequently incubated with PBS containing 3% bovine serum albumin (BSA) at RT for 1 h. Next, the sections were incubated overnight at 4°C with primary antibodies (refer to Table S3 for details). After being rinsed with PBS, the sections were further incubated at RT for 2 h with fluorophore‐conjugated secondary antibodies (listed in Table S3). Finally, the nuclei were stained with 4′,6‐damidino‐2‐phenylindole (DAPI). For combined FISH and immunofluorescence (IF) detection, the cryosections were thoroughly washed after FISH was completed. Then, the primary antibodies and fluorophore‐conjugated secondary antibodies were applied to an additional round of incubation as per the aforementioned procedure. Once again, the nucleus was stained with DAPI. The resulting images were obtained via a fluorescence microscope (Nikon, Japan).

### 2.6 Western Blotting (WB)

Total protein was extracted from tissues and cells via radio immunoprecipitation assay (RIPA) lysis buffer (BOSTER) containing a cocktail of protease and phosphatase inhibitors (Selleck). The protein concentration was quantified via a BCA protein assay kit (Beyotime, China). The samples were then mixed with an equal volume of urea buffer and heated at 65°C for 15 min. Equal amounts of sample protein were electrophoresed on sodium dodecyl‐sulfate polyacrylamide gel electrophoresis (SDS‐PAGE) gels and subsequently transferred to polyvinylidene fluoride (PVDF) membranes (Millipore, USA). The membranes were blocked in Tris‐buffered solution containing 5% skim milk and 0.1% Tween‐20 at RT for 1 h. The membranes were subsequently incubated overnight at 4°C with primary antibodies (listed in Table S3). After three washes, the membranes were incubated at RT for 2 h with secondary antibodies (listed in Table S3). Finally, the visualization of the membranes was achieved through NcmECL Ultra (NCM Biotech, China), followed by signal detection via a Fusion Solo Imaging System (VIBER LOURMAT, France).

### 2.7 Human Mast Cell Culture and Treatment

The human mast cell line LUVA was obtained from MeisenCTCC (MEISEN CELL, China). LUVA cells are immortalized human mast cells that can be maintained without stem cell factor (SCF) and normally display KIT signaling [20]. The cells were cultured in StemPro‐34 SFM (Thermo Fisher Scientific) containing 1 × GlutaMAX (Gibco), 100 U/mL penicillin and 100 mg/mL streptomycin (Gibco). Mast cells were treated with 0, 10, 25, or 50 ng/mL IL‐33 (Sigma) or SCF (MedChemExpress) at concentrations of 0, 10, 100, or 1000 ng/mL, respectively. In addition, the cells were also exposed to 10 ng/mL IL‐33 for 6, 12, 24, or 48 h.

### 2.8 Mimics/Inhibitor Preparation and Cell Transfection

The miR‐221‐3p mimic, inhibitor, and negative control were synthesized by GenePharma (China; the sequences are listed in Table S4). Mimics were transfected into human mast cells at a concentration of 10 nM, while the mimic negative control (m‐NC) was transfected at the same concentration. The inhibitor was transfected at a concentration of 50 nM, and the inhibitor negative control (i‐NC) was also transfected at a concentration of 50 nM. Transfection was carried out via jetPRIME transfection reagent (Polyplus, France) for 24 h. Following transfection, several subsets of mast cells were stimulated with 10 ng/mL IL‐33 for 6–24 h. The cells were subsequently harvested for RNA isolation and protein extraction.

### 2.9 Lentiviral Plasmid Preparation and Cell Transduction

Short hairpin RNAs (shRNAs) targeting KIT (sh‐KIT) and control shRNAs (sh‐CTL) were designed by GeneCopoeia (Maryland, USA) and cloned and inserted into the lentiviral transfer vector psi‐LVRU6GP. The lentiviral transfer vector was cotransfected into HEK293T cells with Lenti‐Pac™ HIV packaging mix (GeneCopoeia) to obtain purified lentivirus particles. Subsequently, lentiviral transduction of mast cells was performed as previously described [21]. After selection with puromycin, the cells were further refined through fluorescence activated cell sorting (FACS) on the basis of the quantification of enhanced green fluorescent protein (eGFP). Consequently, a lentiviral system for knocking down KIT expression in human mast cells was established. These cells were then either stimulated with IL‐33 or transiently transfected with a miR‐221‐3p inhibitor followed by IL‐33 stimulation.

### 2.10 Cell Proliferation

A Cell Counting Kit‐8 (CCK‐8, Oriscience, China) was used to assess the growth of mast cells. Human mast cells were seeded at a density of 5000 cells per well in 96‐well plates in the presence or absence of 100 ng/mL SCF. Following incubation for 1, 2, and 3 days, the cells were treated with 10% CCK‐8 in StemPro‐34 SFM for 1 h at 37°C. Subsequently, absorbance readings were taken at a wavelength of 450 nm using a multiscan spectrum (BioTek). Additionally, sh‐CTL mast cells and sh‐KIT mast cells were plated at a density of 5000 cells per well in 96‐well plates and incubated under standard culture conditions. After intervals of 1, 2, 3, and 4 days, the cells were treated with 10% CCK‐8 for 1 h, and then, absorbance readings were taken at a wavelength of 450 nm via a multiscan spectrum.

### 2.11 Cell Apoptosis Analysis

An Annexin V‐Alexa Fluor 647/7‐AAD Apoptosis Detection Kit (4A Biotech, China) was used to detect mast cell apoptosis. A single mast cell suspension was made and centrifuged at 1500 rpm for 5 min, followed by washing in cold PBS twice. Then, the cells were resuspended in 1 × binding buffer, and the cell density was adjusted to 1–5 × 10^6^/mL. A total of 100 µL of the cell suspension plus 5 µL of Annexin V Alexa Fluor 647 was mixed and incubated at RT for 5 min. After the addition of 10 µL of 7‐AAD and 400 µL of PBS, the mixture was detected immediately. The percentage of apoptotic cells was analyzed via a dual laser flow cytometer (CytoFLEX, Beckman) and estimated via FlowJo software.

### 2.12 Bioinformatics Prediction and Dual‐Luciferase Reporter Gene Assay

The target genes of miR‐221‐3p were predicted via public miRNA databases, including TargetScan (www.targetscan.org/), Tarbase (dianalab.e‐ce.uth.gr/tarbasev9), miRDB (mirdb.org/), and miRTarbase (mirtarbase.cuhk.edu.cn/∼miRTarBase/). The wild‐type 3′‐UTR of KIT (KIT‐wt) containing the putative binding site was synthesized and cloned and inserted into the pEZX‐FR02 vector by GeneCopoeia. Additionally, the mutant 3′‐UTR of KIT (KIT‐mut) lacking the miR‐221‐3p‐binding site was constructed and cloned and inserted into the pEZX‐FR02 vector. Subsequently, the HEK293T cells were cotransfected with either the KIT‐wt vector or the KIT‐mut vector along with the miR‐221‐3p mimic. Following a 48‐h incubation period, firefly and *Renilla* luciferase activities were quantified via the Luc‐Pair Duo‐Luciferase HS Assay Kit from GeneCopoeia.

### 2.13 Statistical Analysis

Statistical analyses were conducted via GraphPad Prism (GraphPad Software, USA). The data are presented as the mean ± standard error of the mean. The Mann–Whitney *U*‐test and the Kruskal–Wallis test were used for comparisons between two groups and among multiple groups in nasal tissues, respectively. Unpaired *t*‐tests and one‐way analysis of variance (ANOVA) were carried out to compare the differences in the in vitro data. The Spearman correlation coefficient was used to determine variable relationships. Asterisks denote statistical significance (*p < 0.05, **p < 0.01, ***p < 0.001).

## 3 Results

### 3.1 The Expression and Location of miR‐221‐3p in CRSwNP

**FIGURE 1:**
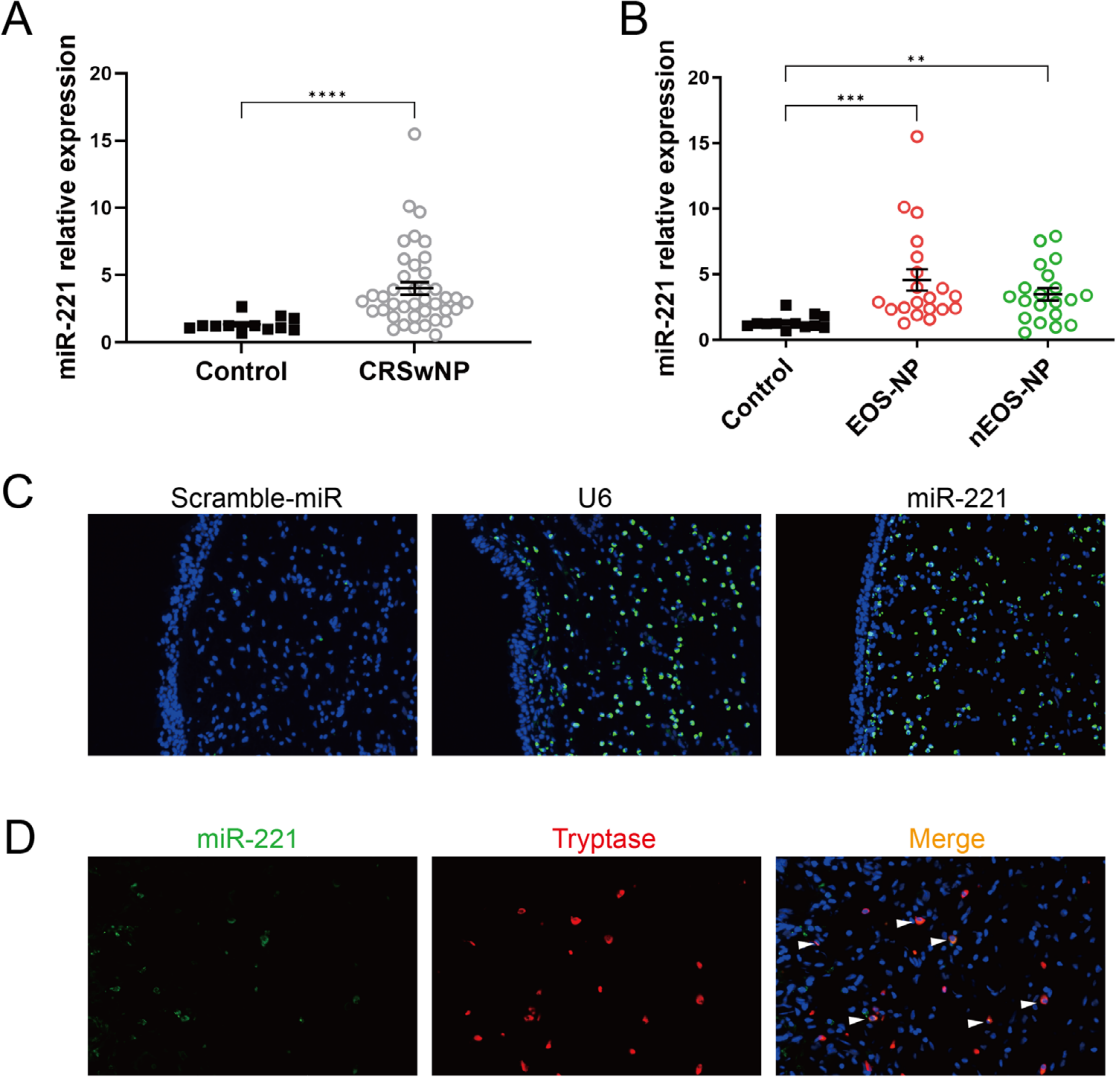
The expression and localization of miR‐221‐3p in chronic rhinosinusitis with nasal polyps (CRSwNP). (A) The expression levels of miR‐221‐3p were compared between the control group (*n* = 12) and the CRSwNP group (*n* = 40) via a real‐time quantitative polymerase chain reaction (qPCR) assay. The Mann–Whitney *U*‐test was used for comparisons between two groups. (B) The expression of miR‐221‐3p was analyzed via a qPCR assay in the control group (*n* = 12), EOS‐NP group (*n* = 20), and nEOS‐NP group (*n* = 20). The Kruskal–Wallis test was used for comparisons among multiple groups. (C) Representative fluorescence in situ hybridization (FISH) images demonstrating the expression of miR‐221‐3p in CRSwNP. Scramble‐miR and U6 were used as negative control or positive control, respectively. Green fluorescence indicates the presence of miRNAs. (D) Representative graph showing costaining of miR‐221‐3p (green) with tryptase (red) in CRSwNP (white arrows). Asterisks indicate statistical significance, *p < 0.05, **p < 0.01, ***p < 0.001.

To validate the elevated expression levels of miR‐221‐3p in CRSwNP, a qPCR assay was performed on 12 control subjects and 40 patients with CRSwNP. QPCR analysis revealed significant upregulation of miR‐221‐3p in the CRSwNP group compared with the control group (*p* < 0.001; Figure 1A). Upon classification of CRSwNP into eosinophilic CRSwNP (EOS‐NP) and noneosinophilic CRSwNP (nEOS‐NP), the qPCR results revealed an increase in miR‐221‐3p expression in both the EOS‐NP and nEOS‐NP groups compared with the control group, although no significant difference was observed between the EOS‐NP and nEOS‐NP groups (Figure 1B). Subsequent investigation of miR‐221‐3p expression in tissues through FISH revealed its detectable presence within CRSwNP (Figure 1C). Furthermore, the cell distribution of miR‐221‐3p was examined by costaining combined with miRNA in situ hybridization and immunohistochemical detection of protein markers, which revealed the coexpression of miR‐221‐3p with the mast cell marker tryptase in CRSwNP (Figure 1D and Supporting Information Figure 1).

### 3.2 Mast Cell Expression and Distribution in CRSwNP

In light of the intimate correlation between mast cells and type 2 inflammation, we conducted IF staining to describe the expression and distribution of tryptase, a mast cell marker, in CRSwNP. The findings revealed the presence of mast cells in both the control group and the CRSwNP group (Supporting Information Figure 2A–C). The quantification of mast cell numbers in HPFs revealed a greater presence of mast cells in the eosinophilic CRSwNP group than in the control and noneosinophilic CRSwNP groups (Supporting Information Figure 2D,E). Furthermore, our observations revealed that mast cells were capable of breaching the basement membrane and infiltrating the epithelium in eosinophilic CRSwNP (Supporting Information Figure 2B), whereas this phenomenon was not observed in the control group or noneosinophilic CRSwNP (Supporting Information Figure 2A,C).

### 3.3 Correlations Between miR‐221‐3p and Type 2 Cytokines in CRSwNP

**FIGURE 2:**
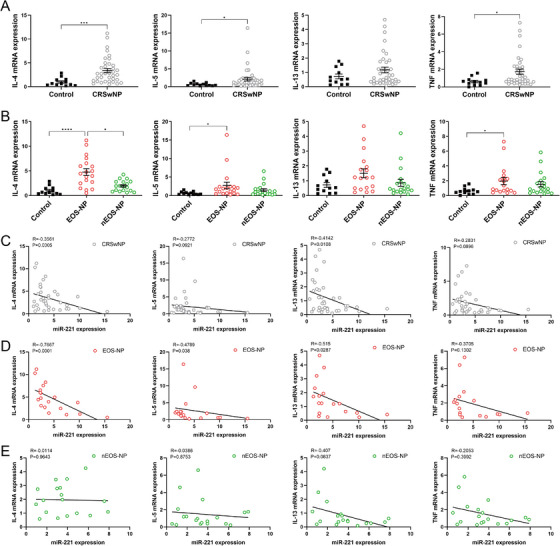
Correlations between miR‐221‐3p and type 2 cytokines in chronic rhinosinusitis with nasal polyps (CRSwNP). (A) The mRNA expression of interleukin (IL)‐4, IL‐5, IL‐13, and tumor necrosis factor (TNF) in the control and CRSwNP groups was analyzed via a real‐time quantitative polymerase chain reaction (qPCR) assay. The Mann–Whitney *U*‐test was used for comparisons between two groups. (B) The mRNA expression of IL‐4, IL‐5, IL‐13, and TNF in the control, EOS‐NP, and nEOS‐NP groups was analyzed via a qPCR assay. The Kruskal–Wallis test was used for comparisons among the three groups. The correlations between miR‐221‐3p and IL‐4, IL‐5, IL‐13, and TNF were investigated within different groups: (C) CRSwNP, (D) EOS‐NP, and (E) nEOS‐NP. *R* values indicate Spearman correlation coefficients.

The mRNA expression levels of IL‐4, IL‐5, IL‐13, and TNF were evaluated via qPCR in the control group and CRSwNP groups. The expression levels of IL‐4, IL‐5, and TNF in CRSwNP were significantly elevated compared with those in the control group (Figure 2A), whereas the expression levels of IL‐4, IL‐5, and TNF were greater significance in the eosinophilic CRSwNP group than in the control group (Figure 2B).

To investigate the potent role of miR‐221‐3p, we examined the correlations between miR‐221‐3p and IL‐4, IL‐5, IL‐13, and TNF in CRSwNP. The Pearson correlation test revealed a significant negative correlation between miR‐221‐3p and both IL‐4 and IL‐5 in CRSwNP (Figure 2C). We subsequently investigated the correlations between miR‐221‐3p and cytokines in noneosinophilic CRSwNP and eosinophilic CRSwNP separately. The Pearson correlation test indicated a significant negative correlation between miR‐221‐3p and IL‐4, IL‐5, and IL‐13 in eosinophilic CRSwNP but not in noneosinophilic CRSwNP (Figure 2D,E). Consequently, we speculated that upregulated miR‐221‐3p can exert a negative regulatory effect on type 2 inflammation in CRSwNP.

### 3.4 Effects of miR‐221‐3p on Cytokine Expression and Signaling Pathways in Human Mast Cells in the Presence and Absence of IL‐33

We then investigated whether miR‐221‐3p could regulate the expression of IL‐4, IL‐5, IL‐13, IL‐1β, IL‐6, and TNF in mast cells stimulated with IL‐33. To evaluate the optimal dose and time response of IL‐33, we measured the expression of type 2 and proinflammatory cytokines at different concentrations and time points. The results revealed that treatment with 10 ng/mL IL‐33 for 6 h significantly increased the mRNA expression of IL‐5, IL‐13, and TNF (Supporting Information Figure 3B–D, F–H) but not that of IL‐4 (Supporting Information Figure 3A,E). Interestingly, the stimulatory effect of IL‐33 on cytokine expression decreased over time. Additionally, stimulation with IL‐33 did not impact the expression of IL‐1β or IL‐6 (data not shown).

**FIGURE 3:**
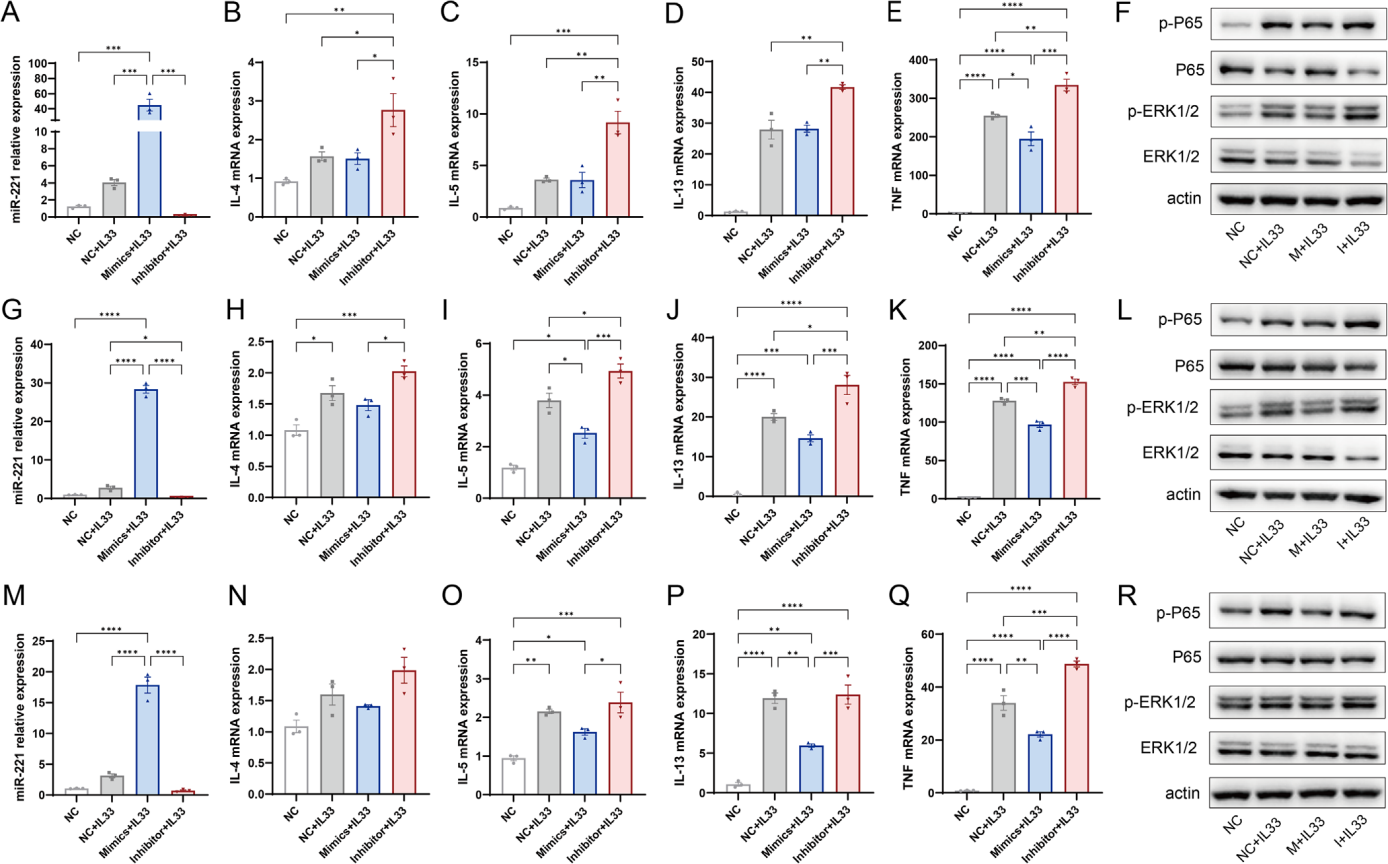
Effect of miR‐221‐3p on cytokine expression and signaling pathways in mast cells induced by interleukin (IL)‐33. Expression of miR‐221‐3p, IL‐4, IL‐5, IL‐13, and tumor necrosis factor (TNF) in mast cells transfected with miR‐221‐3p and treated with IL‐33 for 6 h (A–E), 12 h (G–K), or 24 h (M–Q) was analyzed via a real‐time quantitative polymerase chain reaction (qPCR) assay. One‐way analysis of variance (ANOVA) was used for comparisons among the four groups. The activation of ERK and P65 was determined by western blotting (WB) in mast cells transfected with miR‐221‐3p followed by treatment with IL‐33 for 6 h (F), 12 h (L), or 24 h (R). A representative western blotting (WB) result from three independent experiments is shown.

Then, we conducted miR‐221‐3p oligo transfection followed by treatment with 10 ng/mL IL‐33 for 6, 12, or 24 h. We initially confirmed that the mimics and inhibitor significantly increased or inhibited the expression of miR‐221‐3p in mast cells, respectively (Figure 3A,G,M and Supporting Information Figure 4A). At 6 h, the miR‐221‐3p inhibitor increased the mRNA expression of IL‐4, IL‐5, IL13, and TNF, whereas the miR‐221‐3p mimic inhibited only TNF mRNA expression (Figure 3B–E). Additionally, the miR‐221‐3p mimic and inhibitor inhibited or promoted the activation of the ERK1/2 and P65 pathways, respectively (Figure 3F). After 12 h, the miR‐221‐3p inhibitor led to an increase in IL‐5, IL‐13, and TNF mRNA expression; conversely, the miR‐221‐3p mimic inhibited IL‐5, IL‐13, and TNF mRNA expression (Figure 3I–K). Furthermore, similar to the findings at 6 h, both the miR‐221‐3p mimic and inhibitor inhibited or promoted the activation of the ERK1/2 and P65 pathways, respectively (Figure 3L). Finally, the miR‐221‐3p inhibitor increased only TNF mRNA expression at 24 h, but the miR‐221‐3p mimic was still able to inhibit the expression of IL‐5, IL‐13, and TNF mRNAs (Figure 3O–Q). Moreover, only the miR‐221‐3p mimic inhibited the activation of the ERK1/2 and P65 pathways (Figure 3R).

Notably, we observed that treatment with IL‐33 induced the expression of miR‐221‐3p in mast cells (Figure 3A,G,M). We subsequently investigated the effect of miR‐221‐3p on mast cells. Our findings demonstrated that the transfection of miR‐221‐3p mimic or inhibitor did not affect the mRNA expression of IL‐4, IL‐5, IL13 or TNF in mast cells (Supporting Information Figure 4B–E) nor affect the activation of ERK1/2 and P65 (Supporting Information Figure 4F). These results indicate that miR‐221‐3p can be induced by IL‐33 in mast cells and plays a negative regulatory role in cytokine expression and signaling pathways in IL‐33‐induced mast cell activation.

### 3.5 MiR‐221‐3p Directly Targets KIT

**FIGURE 4:**
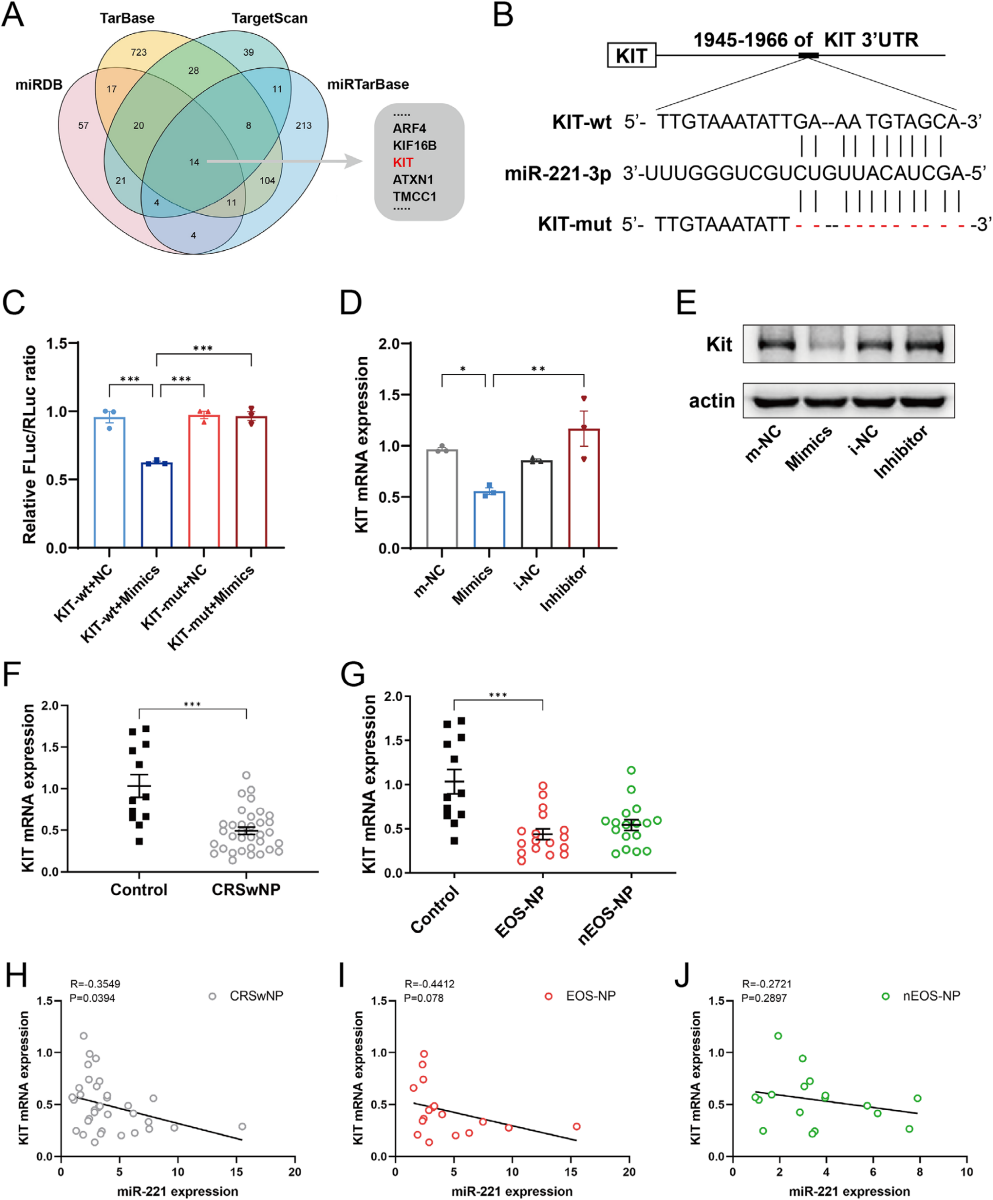
MiR‐221‐3p directly targets KIT. (A) Venn diagram illustrating the target genes of miR‐221‐3p identified from the TargetScan, miRDB, miRTarBase, and TarBase databases. (B) Predicted binding sites of miR‐221‐3p within the 3′‐UTR of KIT mRNA are shown. (C) A dual‐luciferase activity assay was performed in HEK293T cells after cotransfection with miR‐221‐3p negative control/mimic and plasmids containing KIT‐wt or KIT‐mut, and the ratio of FLuc to RLuc was determined. (D) The mRNA expression levels of KIT were analyzed via a real‐time quantitative polymerase chain reaction (qPCR) assay in mast cells transfected with the miR‐221‐3p oligo for 24 h. (E) KIT protein expression was evaluated through western blotting (WB) in mast cells transfected with the miR‐221‐3p oligo for 24 h. A representative WB result from three independent experiments is shown. (F, G) The mRNA expression of KIT in the control group and the chronic rhinosinusitis with nasal polyps (CRSwNP) groups was analyzed via a qPCR assay. (H–J) The correlations between miR‐221‐3p and KIT were investigated in CRSwNP groups. *R* values indicate Spearman correlation coefficients.

We aimed to elucidate the mechanisms by which miR‐221‐3p inhibits the transcription of IL‐5, IL‐13, and TNF in IL‐33‐stimulated mast cells. To predict the target genes of miR‐221‐3p, we utilized public miRNA databases, including TargetScan, miRDB, miRTarBase, and TarBase, to identify potential binding genes for miR‐221‐3p. A total of 14 target genes were identified (Figure 4A). Among these targets, KIT is known to be necessary for IL‐33‐induced cytokine production in bone marrow‐derived murine mast cells [22]. Sequence analysis revealed that miR‐221‐3p can interact with sites 1945–1966 in the 3′UTR of KIT mRNA (Figure 4B). A sequence containing potential binding sites was inserted into the cloning vector pEZX‐FR02 as a wild type (KIT‐wt), whereas a sequence with knockout binding sites was inserted as a mutated type (KIT‐mut). A luciferase reporter assay was subsequently performed to assess the effect of miR‐221‐3p on KIT expression in HEK293T cells. Compared with cotransfection with the negative control, cotransfection of KIT‐wt with mimics resulted in a significant reduction in relative luciferase activity, whereas the luciferase activity remained unaffected when the binding sites within the KIT 3′‐UTR were mutated (Figure 4C). We further evaluated the effect of miR‐221‐3p on KIT expression in human mast cells. The results indicated that upregulation of miR‐221‐3p could significantly suppress both KIT mRNA and protein expression levels (Figure 4D,E). These findings confirm that KIT is indeed a direct target gene of miR‐221‐3p in mast cells.

**FIGURE 5:**
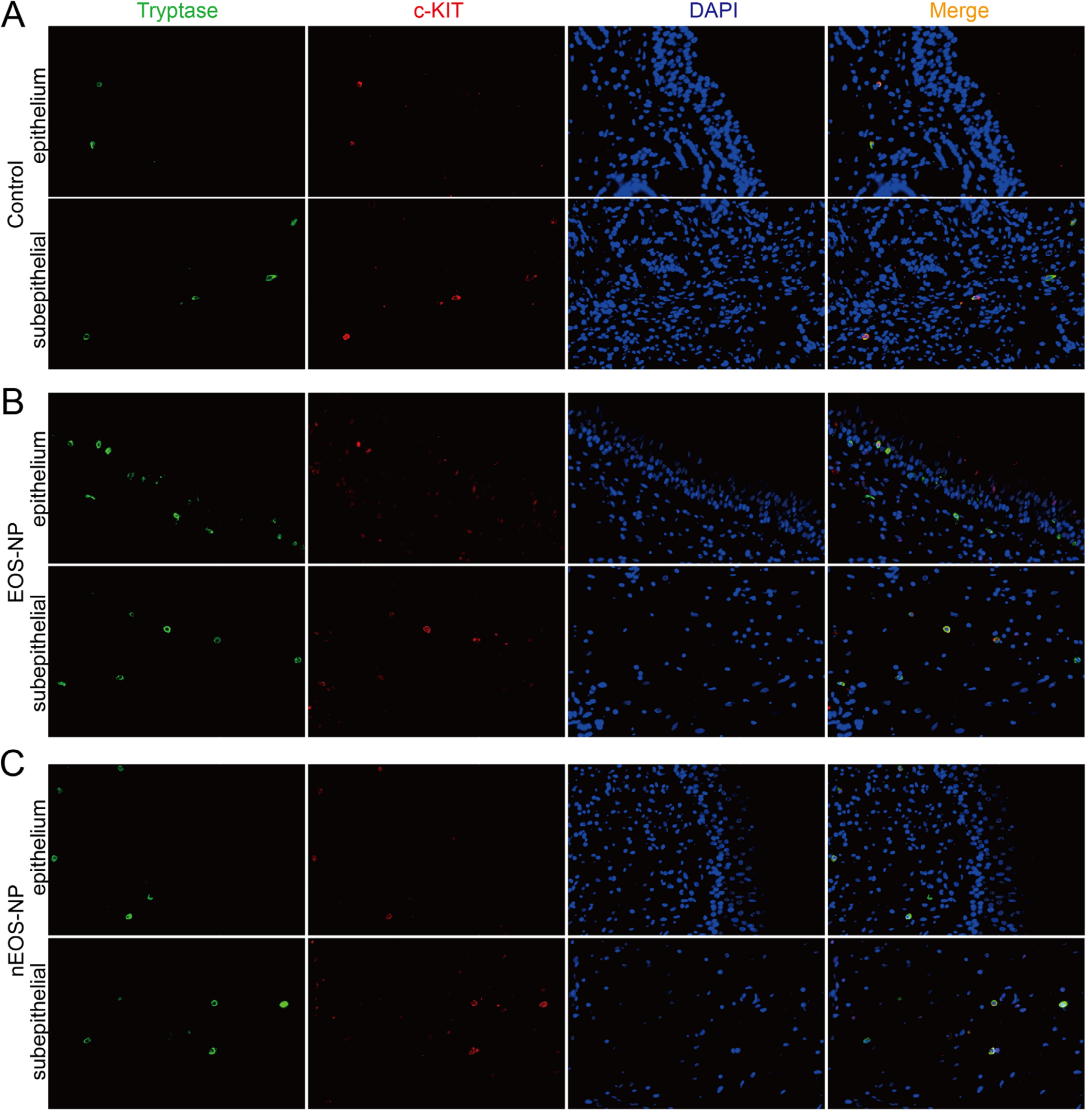
Double immunostaining of KIT and tryptase in nasal tissues. (A) Representative immunofluorescence (IF) images showing the costaining of KIT (red) and tryptase (green) in the lamina propria of the control group. (B) Representative IF images demonstrating the costaining of KIT (red) and tryptase (green) in the epithelium and subepithelial tissue of eosinophilic chronic rhinosinusitis with nasal polyps (CRSwNP). (C) Representative IF images showing the costaining of KIT (red) and tryptase (green) in the lamina propria of noneosinophilic CRSwNP.

Next, we investigated the expression of KIT in CRSwNP and its correlation with miR‐221‐3p. The qPCR assay revealed a reduction in KIT mRNA expression levels in both CRSwNP and eosinophilic CRSwNP (Figure 4F,G). Pearson correlation analysis revealed a negative association between miR‐221‐3p expression levels and KIT mRNA expression in CRSwNP (Figure 4H–J). Furthermore, we conducted double IF staining to examine the expression of KIT in mast cells from the control and CRSwNP groups. The results demonstrated that KIT signals were present in all mast cells of the control group (Figure 5A) and in most mast cells of noneosinophilic CRSwNP (Figure 5C). However, many mast cells of eosinophilic CRSwNP did not exhibit detectable KIT signals, particularly intraepithelial mast cells and those located near the epithelium within the lamina propria (Figure 5B). In summary, our findings suggest that miR‐221‐3p may suppress the expression of KIT by binding to its mRNA within the mast cells of CRSwNP.

### 3.6 KIT Regulates Type 2 Cytokine and TNF Expression Induced by IL‐33 in Human Mast Cells

**FIGURE 6:**
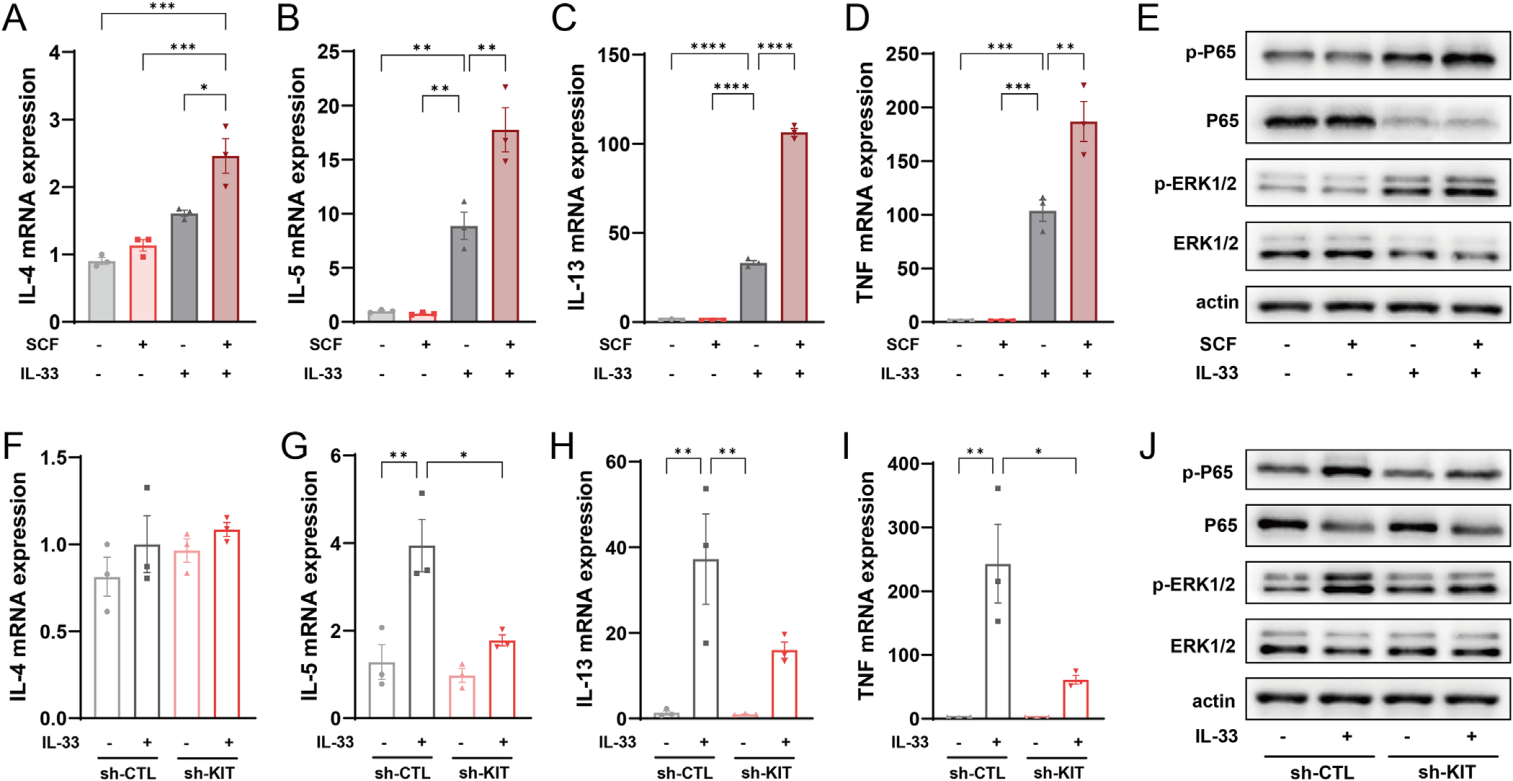
The expression of cytokines and the activation of signals induced by interleukin (IL)‐33 in human mast cells are regulated by KIT. The mRNA expression levels of (A) IL‐4, (B) IL‐5, (C) IL‐13, and (D) tumor necrosis factor (TNF) were quantified via a real‐time quantitative polymerase chain reaction (qPCR) assay in mast cells that were stimulated with IL‐33 (10 ng/mL, 6 h) following pretreatment with stem cell factor (SCF; 100 ng/mL, 1 h). (E) P65 and ERK activation in mast cells under the same conditions was determined via western blotting (WB). A representative WB result from three independent experiments is shown. A qPCR assay was used to assess the mRNA expression of (F) IL‐4, (G) IL‐5, (H) IL‐13, and (I) TNF in sh‐CTL and sh‐KIT mast cells with or without treatment with IL‐33 (10 ng/mL, 6 h). (J) P65 and ERK activation was determined by WB in sh‐CTL and sh‐KIT mast cells under the same conditions. A representative WB result from three independent experiments is shown.

The differentiation, survival, and proliferation of mast cells are mediated by the activation of KIT induced by SCF [23]. Therefore, we investigated the role of KIT activation in signaling pathway activation and cytokine expression in LUVA cells. Treatment with 100 ng/mL SCF increased the proliferation of human mast cells (Supporting Information Figure 5A). However, treatment with SCF alone did not increase the expression of cytokines or induce the activation of ERK1/2 and P65, which remained unaffected by the treatment concentration (Supporting Information Figure 5B–F). We subsequently prestimulated mast cells with SCF prior to treatment with IL‐33. Compared with stimulation with IL‐33 alone, SCF promoted IL‐33‐induced cytokine mRNA expression (Figure 6A–D). Additionally, SCF was observed to enhance the IL‐33‐induced activation of ERK1/2 and P65 (Figure 6E).

Next, we evaluated the effects of KIT downregulation on cytokine expression and signaling pathway activation in mast cells. The lentiviral particles carrying the KIT shRNA sequence were transduced into human mast cells to inhibit KIT expression. A significant decrease in KIT expression was observed in mast cells transduced with the sh‐KIT vector compared with those transduced with the sh‐CTL vector (Supporting Information Figure 6A,D,I), indicating successful construction of KIT‐knockdown mast cells. Furthermore, we demonstrated that the knockdown of KIT did not have an obvious effect on LUVA cell proliferation and apoptosis (Supporting Information Figure 6B,C), the mRNA expression of the cytokines IL‐4, IL‐5, IL‐13, and TNF (Supporting Information Figure 6E–H), or the activation of ERK1/2 and P65 (Supporting Information Figure 6I). Then, KIT‐knockdown mast cells were stimulated with 10 ng/mL IL‐33 for 6 h. The mRNA expression of IL‐5, IL‐13, and TNF was significantly lower in KIT‐knockdown mast cells than in those transduced with the sh‐CTL vector (Figure 6G–I). Moreover, ERK1/2 and P65 activation was also repressed in KIT‐knockdown mast cells compared with those transduced with the sh‐CTL vector (Figure 6J). These findings suggest that KIT is essential for the effective response of mast cells to IL‐33 stimulation.

### 3.7 MiR‐221‐3p Regulates IL‐33‐Dependent Cytokine Expression Through Targeting KIT in Mast Cells

**FIGURE 7:**
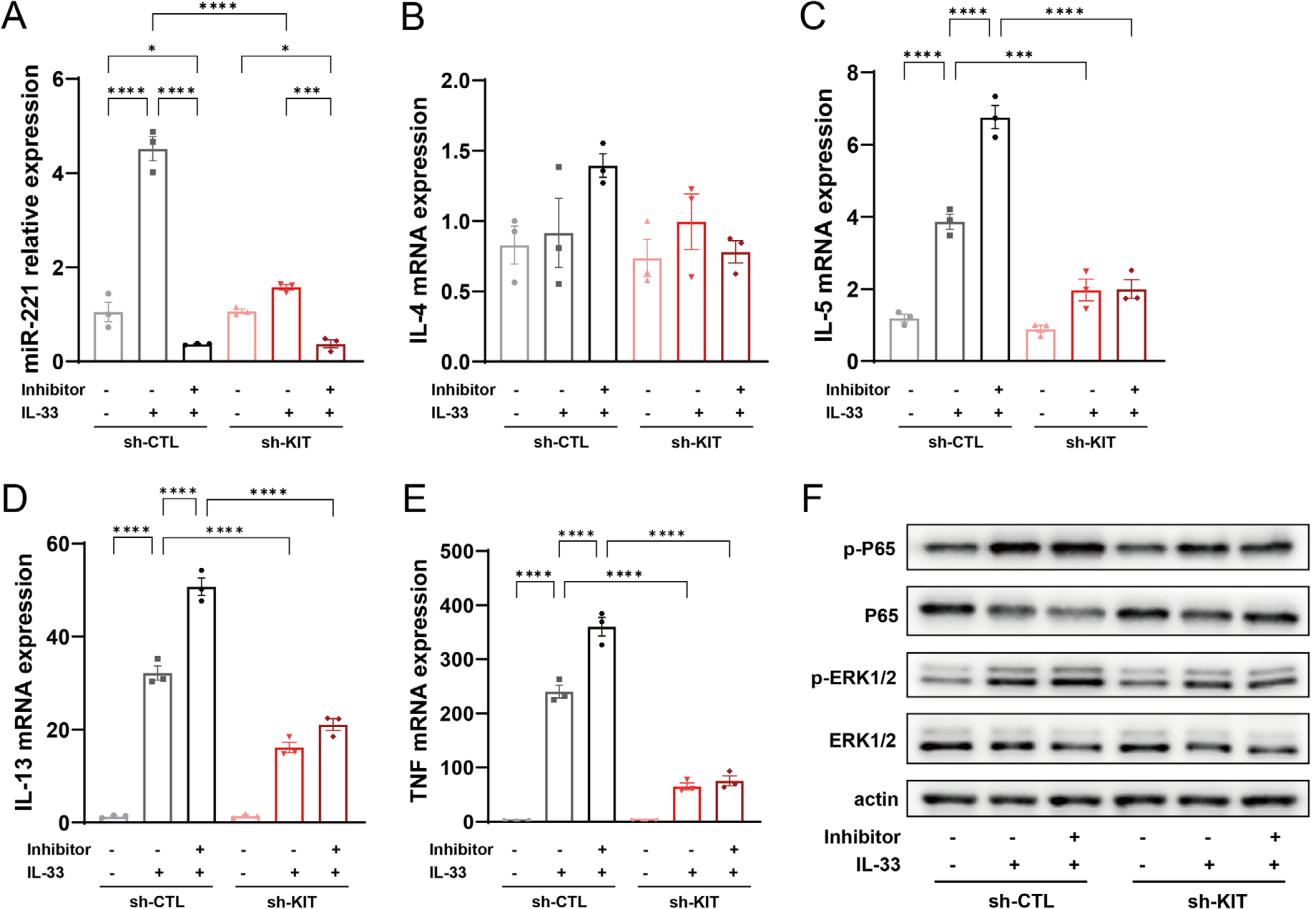
MiR‐221‐3p regulates interleukin (IL)‐33‐dependent cytokine expression and signal activation through targeting KIT in mast cells. After transfection with the negative control oligo or miR‐221‐3p inhibitor for 24 h, both the sh‐CTL and sh‐KIT mast cells were stimulated with IL‐33 (10 ng/mL, 6 h). Mast cells without miR‐221‐3p inhibitor transfection and IL‐33 treatment were used as the control group. The expression of (A) miR‐221‐3p, (B) IL‐4, (C) IL‐5, (D) IL‐13, and (E) tumor necrosis factor (TNF) was analyzed via a real‐time quantitative polymerase chain reaction (qPCR) assay. One‐way analysis of variance (ANOVA) was used for comparisons among the six groups. (F) P65 and ERK activation in mast cells was determined via western blotting (WB). A representative WB result from three independent experiments is shown.

We then investigated whether miR‐221‐3p regulates the expression of cytokines through KIT in IL‐33‐stimulated mast cells. The miR‐221‐3p inhibitor was transfected into sh‐CTL mast cells and sh‐KIT mast cells, which were then stimulated with IL‐33 to assess the role of KIT knockdown in the promoting effect of the miR‐221‐3p inhibitor. As expected, in sh‐CTL mast cells, stimulation with IL‐33 increased the expression of miR‐221‐3p, IL‐5, IL‐13, and TNF, and the miR‐221‐3p inhibitor significantly reduced the expression of miR‐221‐3p but increased the mRNA expression of IL‐5, IL‐13, and TNF (Figure 7A–E). In addition, IL‐33 stimulation promoted the activation of ERK1/2 and P65, which was further enhanced by the miR‐221‐3p inhibitor (Figure 7F). In KIT‐knockdown mast cells, the stimulatory effect of IL‐33 treatment on the expression of miR‐221‐3p and cytokines, as well as ERK1/2 and P65 activation, was significantly reduced. Additionally, the enhancing effect of the miR‐221‐3p inhibitor on IL‐33 treatment was also abolished (Figure 7A–F). These results suggest that miR‐221‐3p regulates cytokine expression and signaling pathway activation by targeting KIT in IL‐33‐activated mast cells.

## 4 Discussion

**FIGURE 8:**
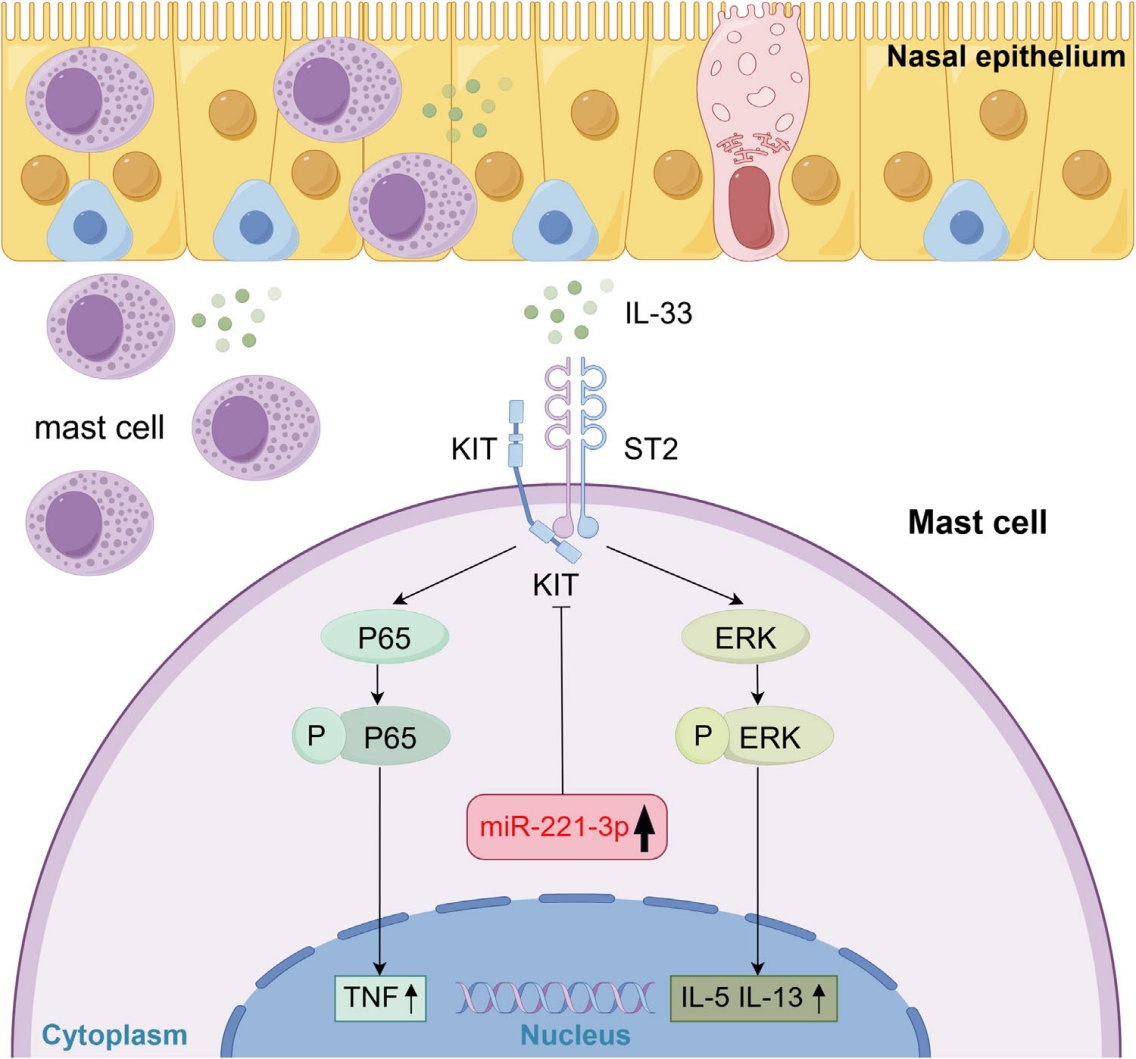
Schematic summary of the anti‐inflammatory role of miR‐221‐3p. In chronic rhinosinusitis with nasal polyps (CRSwNP), nasal epithelial cell‐derived interleukin (IL)‐33 can induce IL‐5, IL‐13, and tumor necrosis factor (TNF) expression by activating P65 and ERK in mast cells. Moreover, IL‐33 can upregulate miR‐221‐3p expression. Elevated miR‐221‐3p may inhibit KIT expression to suppress the activation of IL‐33 signaling.

In the present study, we observed an increase in the expression of miR‐221‐3p and the number of mast cells in CRSwNP compared with those in control subjects. This investigation revealed that miR‐221‐3p mainly is expressed in mast cells in CRSwNP. Our findings suggest that the upregulation of miR‐221‐3p may suppress IL‐33‐induced ERK1/2 and P65 activation by targeting KIT in mast cells, thereby limiting the expression of type 2 cytokines and TNF. These results indicate that miR‐221‐3p may function as a negative regulator of type 2 inflammation mediated by the epithelial cytokine IL‐33 in activated mast cells (as illustrated in Figure 8).

European guidelines classified CRSwNP as eosinophilic airway disease because eosinophilia and elevation of type 2 cytokines are dominant in CRSwNP in Western countries [18]. However, about 30% of CRSwNP in East Asian countries present as noneosinophilic CRSwNP [24]. Therefore, further classification of CRSwNP may be necessary to reflect the intrinsic characteristics of this condition in Asia. Our study confirmed that the expression of miR‐221‐3p was increased in noneosinophilic CRSwNP and eosinophilic CRSwNP compared with that in the control group in western China. This finding is consistent with the results of two other studies, one conducted in northern China [16] and another in Brazil [17]. These findings suggest that geographic variability and ethnic differences may have minimal influences on the expression of miR‐221‐3p, different from the impact on the percentage of eosinophilic versus noneosinophilic CRSwNP. MiR‐221‐3p may play a dynamic role in inflammatory responses, exhibiting both proinflammatory and anti‐inflammatory effects [25, 26]. Notably, we observed a negative correlation between miR‐221‐3p and IL‐4, IL‐5 and IL‐13 in CRSwNP, especially in eosinophilic CRSwNP. Zhang et al. also reported an inverse correlation between epithelial miR‐221‐3p levels and eosinophils in induced sputum and bronchial biopsies, blood eosinophils, and the epithelial gene signature of type 2 status in asthma [27]. Furthermore, our study revealed the presence of miR‐221‐3p within the mast cells of CRSwNP. Therefore, it is reasonable to speculate that elevated miR‐221‐3p may act as a negative regulator of the expression of type 2 cytokines in mast cells, thereby restricting type 2 inflammation in CRSwNP.

A recent study identified four subtypes of mast cells in CRSwNP, all of which express tryptase [11]. In this study, the number of mast cells increased only in eosinophilic CRSwNP, with no significant difference between noneosinophilic CRSwNP and the healthy mucosa. Additionally, mast cells were present only in the epithelium of eosinophilic CRSwNP but did not infiltrate the epithelium of either the control group or noneosinophilic CRSwNP. These findings are similar to those of studies conducted in the United States. However, Baba et al. reported evidence of epithelial infiltration by mast cells in noneosinophilic CRSwNP as well, albeit to a greater extent in eosinophilic CRSwNP [28]. We speculated that this discrepancy may be attributable to the stricter definition of eosinophilic CRSwNP (more than 70 eosinophils/HPF). Recent studies have demonstrated that type 2 inflammation and indirect airway hyperresponsiveness in asthma are related to a shift in mast cells from the submucosa to the airway epithelium and that mast cells cooperate with epithelial cells through IL‐33 signaling to regulate type 2 inflammation [29, 30]. Although direct evidence is lacking, our findings and other studies support the involvement of nasal epithelium–mast cell crosstalk in the pathogenesis of CRSwNP [11, 31, 32]. Therefore, further studies are needed to better understand their pivotal role in CRSwNP.

Through in vitro stimulation of human mast cells, we confirmed the unique effect of IL‐33 on the induction of the type 2 cytokines IL‐5 and IL‐13 but not IL‐4. Additionally, the proinflammatory cytokine TNF, rather than IL‐1β or IL‐6, was also induced by IL‐33, which was consistent with previous studies [29, 33]. Some studies have suggested that costimulation with other cytokines or the use of murine mast cells could lead to the induction of IL‐4, IL‐1β, or IL‐6 production by IL‐33 [34].

Here, we present evidence for the first time that stimulation with IL‐33 can upregulate the expression of miR‐221‐3p in mast cells. Currently, few studies have explored the role of miR‐221‐3p in mast cells. Ramon et al. reported that resting mast cells expressed basal levels of miR‐221, which could be transcriptionally upregulated following stimulation with IgE and SCF [35]. In resting mast cells, basal expression of miR‐221 contributes to the regulation of the cell cycle and skeletal organization. In activated mast cells, miR‐221 is involved in the regulation of cytokine production and cell adhesion [35, 36]. Another study revealed that miR‐221‐3p could increase IL‐4 secretion in mast cells through a pathway involving PTEN, p38, and NF‐κB in a murine asthma model [37]. However, our findings suggest that the overexpression of miR‐221‐3p alone does not activate mast cells or promote cytokine transcription, indicating that it is not an activator of mast cells. In contrast, we observed that the activation effect of mast cell‐dependent IL‐33 signaling can be attenuated by the overexpression of miR‐221‐3p; conversely, it can be enhanced by a reduction in miR‐221‐3p. These findings indicate that the upregulation of miR‐221‐3p caused by IL‐33 serves as a negative feedback mechanism for mast cells to limit potential excessive inflammatory responses.

We confirmed that KIT is a direct target gene of miR‐221‐3p through a luciferase reporter system, while the overexpression of miR‐221‐3p can reduce KIT expression in human mast cells. These findings are consistent with the negative correlation between miR‐221‐3p and KIT in CRSwNP and with the loss of the KIT signal in mast cells of eosinophilic CRSwNP. Our study demonstrated that KIT plays an essential role in the response of mast cells to IL‐33 stimulation. Some studies have revealed that interactions between SCF and KIT can significantly increase the number of mast cells in response to IL‐33 stimulation, possibly through the CaN signaling pathway. Specifically, SCF converts CaN‐independent cytokine responses into CaN‐dependent cytokine responses and prolongs the activation of the IL‐33 signaling pathway [23, 38]. Conversely, we observed that knockdown of KIT led to a significant reduction in the response of mast cells to IL‐33 stimulation. In murine mast cells, ST2 constitutively binds to KIT, and this interaction can be activated by IL‐33 and is critical for IL‐33‐induced function [39, 40]. Both IL‐33‐induced signaling and cytokine production are severely impaired in mast cells lacking expression of KIT [41]. Notably, we also found that the activation or knockdown of KIT alone does not affect ERK or P65 activation levels or cytokine transcription levels in human mast cells, suggesting that effector functions mediated by KIT are dependent on IL‐33 stimulation.

In summary, we demonstrate that miR‐221‐3p, which targets KIT, regulates mast cells in response to IL‐33 stimulation and inhibits the expression of type 2 cytokines and TNF. This finding is in line with the negative correlation between miR‐221‐3p and the cytokines IL‐5, IL‐13, and TNF in CRSwNP. KIT plays a crucial role in the proliferation, migration, and survival of mast cells and has emerged as a therapeutic target for type 2 chronic inflammatory diseases [42]. For example, barzolvolimab, a monoclonal antibody against KIT, has been shown to be effective in chronic prurigo and chronic urticaria [42], and imatinib, a KIT inhibitor, can reduce airway hyperresponsiveness in patients with severe asthma through mast cell depletion [43]. These findings suggest that miR‐221‐3p targeting KIT might be a novel approach for treating type 2 airway inflammatory diseases, including CRSwNP. Therefore, our research confirmed that miR‐221‐3p inhibits mast cell‐dependent type 2 inflammatory conditions, rendering it a protective factor in CRSwNP. Elevated miR‐221‐3p can silence mast cells by targeting KIT, thereby emerging as a novel approach for the treatment of CRSwNP.

## Conflicts of Interest

The authors declare no conflicts of interest.

## Supporting information



Supporting Information
