# Supplementary material for: MiR‐221‐3p Attenuates IL‐33‐Induced Mast Cell Cytokine Expression by Targeting KIT
Source: Int Forum Allergy Rhinol. 2025 Mar 25;15(8):837–50. doi: 10.1002/alr.23558 (PMC12315497; doi:10.1002/alr.23558)
Supplement: Supplementary file 1 — Supporting Information [file ALR-15-837-s001.docx]

**Supplementary Material**

**Supplemental Table 1.** Clinical characteristics of patients.

|  | Control | ECRSwNP | nECRSwNP |
| --- | --- | --- | --- |
| Numbers (n) | 12 | 20 | 20 |
| Male/Female (n) | 10/2 | 11/9 | 12/8 |
| Age, median (range) | 35(19-51) | 49(16-67) | 40(16-60) |
| Allergic rhinitis (n) | 0 | 5 | 3 |
| Asthma (n) | 0 | 11 | 1 |
| EOS counts / HPF, average (range) | 0(0-0) | 28.1(14.3-52.3) | 2.9(0-7.7) |

ECRSwNP, eosinophilic chronic rhinosinusitis with nasal polyps; nECRSwNP, non-eosinophilic chronic rhinosinusitis with nasal polyps; SPT, skin prick test; EOS, eosinophils; HPF, high powered field.

**Supplemental Table 2.** The primer sequences used in qPCR.

| Gene | Forward (5’-3’) | Reverse (5’-3’) |
| --- | --- | --- |
| GAPDH | CATCAAGAAGGTGGTGAATC | TCAAAGGTGGAGGAGTGGGC |
| IL-4 | ATGGGTCTCACCTCCCAACT | TCTGTTACGGTCAACTCGGTG |
| IL-5 | TCTACTCATCGAACTCTGCTGA | CCCTTGCACAGTTTGACTCTC |
| IL-13 | CCTCATGGCGCTTTTGTTGAC | TCTGGTTCTGGGTGATGTTGA |
| TNF | GAGGCCAAGCCCTGGTATG | CGGGCCGATTGATCTCAGC |
| KIT | CGTTCTGCTCCTACTGCTTCG | CCCACGCGGACTATTAAGTCT |

**Supplemental Table 3.** Antibodies used for IF and WB analysis.

| Product | Company | Catalog number | Dilution |
| --- | --- | --- | --- |
| **IF** |  |  |  |
| Mouse anti-Tryptase | Dako | M7052 | 1:500 |
| Rabbit anti-KIT | CST | 3074 | 1:400 |
| Goat anti-Rabbit, Alexa Fluor™ 594 | Invitrogen | A11037 | 1:500 |
| Goat anti-mouse, Alexa Fluor™ 594 | Invitrogen | A11032 | 1:500 |
| Goat anti-mouse, Alexa Fluor™ 488 | Invitrogen | A32723 | 1:500 |
| **WB** |  |  |  |
| Rabbit anti-KIT | CST | 3074 | 1:1000 |
| Rabbit anti-p-P65 | CST | 3033 | 1:1000 |
| Rabbit anti-P65 | CST | 8482 | 1:1000 |
| Rabbit anti-p-ERK | CST | 9101 | 1:1000 |
| Rabbit anti-ERK | Zenbio | 343830 | 1:1000 |
| Mouse anti-beta-actin | Huabio | EM21002 | 1:100000 |
| Goat anti-rabbit, HRP | Zenbio | 511203 | 1:10000 |
| Goat anti-mouse, HRP | Zenbio | 511103 | 1:10000 |

IF, immunofluorescence; WB, western blotting.

**Supplemental Table 4.** The mimics, inhibitor and negative control sequences.

| RNA oligo | Sense (5’-3’) | Anti-sense (5’-3’) |
| --- | --- | --- |
| Mimics | AGCUACAUUGUCUGCUGGGUUUC | AACCCAGCAGACAAUGUAGCUUU |
| Negative control | UUCUCCGAACGUGUCACGUTT | ACGUGACACGUUCGGAGAATT |
| Inhibitor | GAAACCCAGCAGACAAUGUAGCU |  |
| Negative control | CAGUACUUUUGUGUAGUACAA |  |


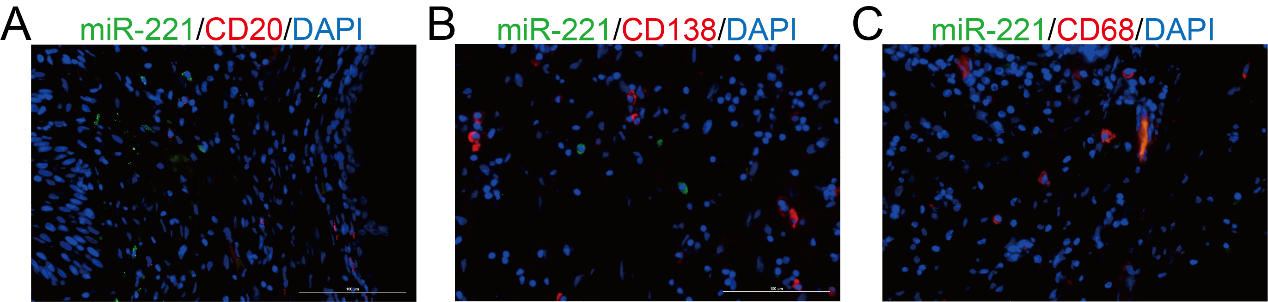
**Supplementary Figure 1**. The celluar localization of miR-221-3p in CRSwNP. Images show co-staining of miR-221-3p (green) with (A) CD20 (red), (B) CD138 (red) and (C) CD68 (red) in CRSwNP.


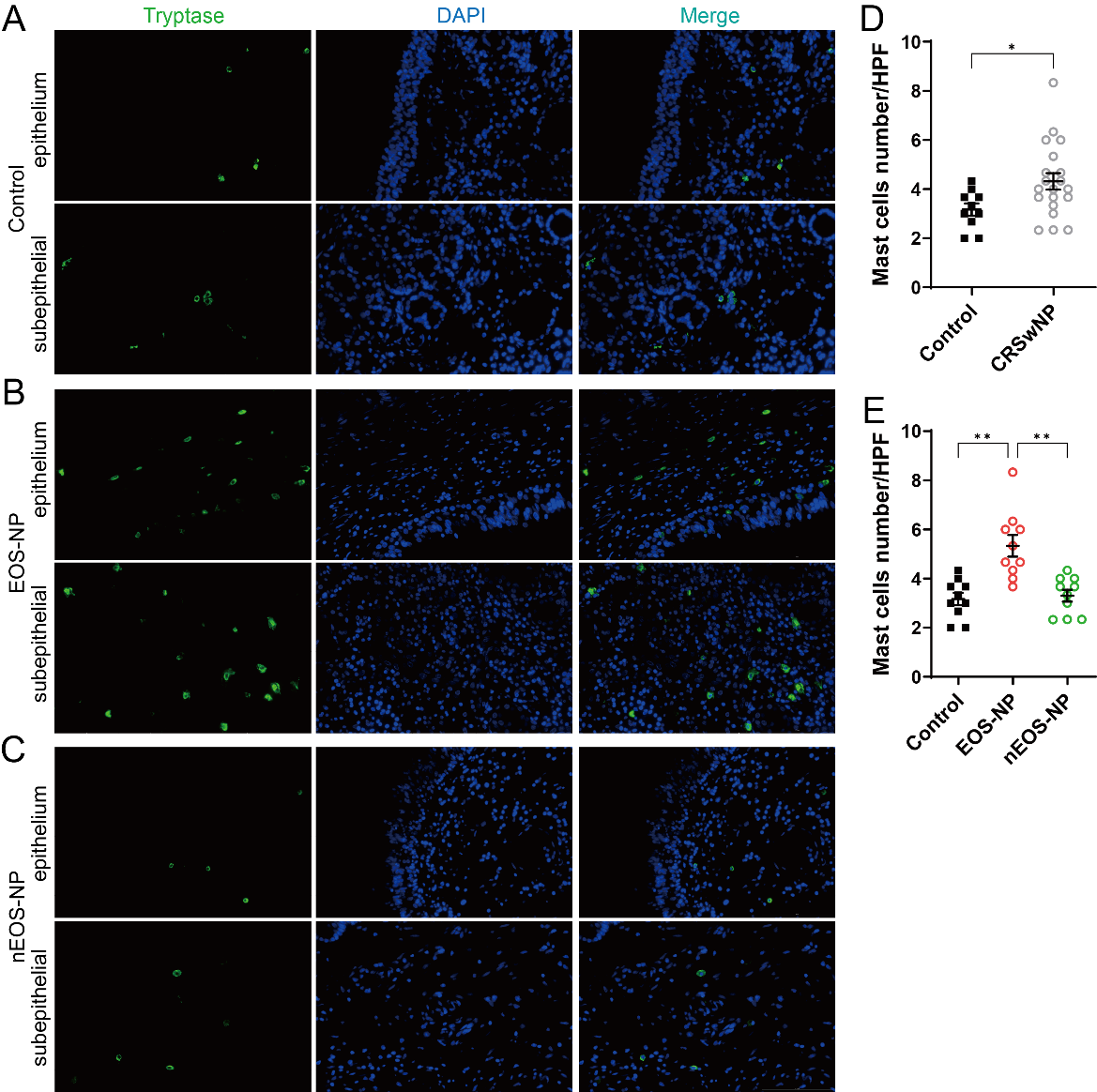
**Supplementary Figure 2**. The expression and distribution of mast cells in CRSwNP. (A) Representative IF images showing the distribution of mast cells (green) in the lamina propria of the control group. (B) Representative IF images demonstrating the distribution of mast cells in the epithelium and subepithelial tissue of eosinophilic CRSwNP. (C) Representative IF images showing the distribution of mast cells in the lamina propria of noneosinophilic CRSwNP. (D) Quantification of tryptase-positive mast cell numbers in tissues from the control group and the CRSwNP group. The Mann‒Whitney U test was used for comparisons between two groups. (E) Quantification of tryptase-positive mast cell numbers in tissues from the control group, eosinophilic CRSwNP group and noneosinophilic CRSwNP group. The Kruskal‒Wallis test was used for comparisons among multiple groups. Asterisks indicate statistical significance, *P<0.05, **P<0.01. HPF, high-powered field.


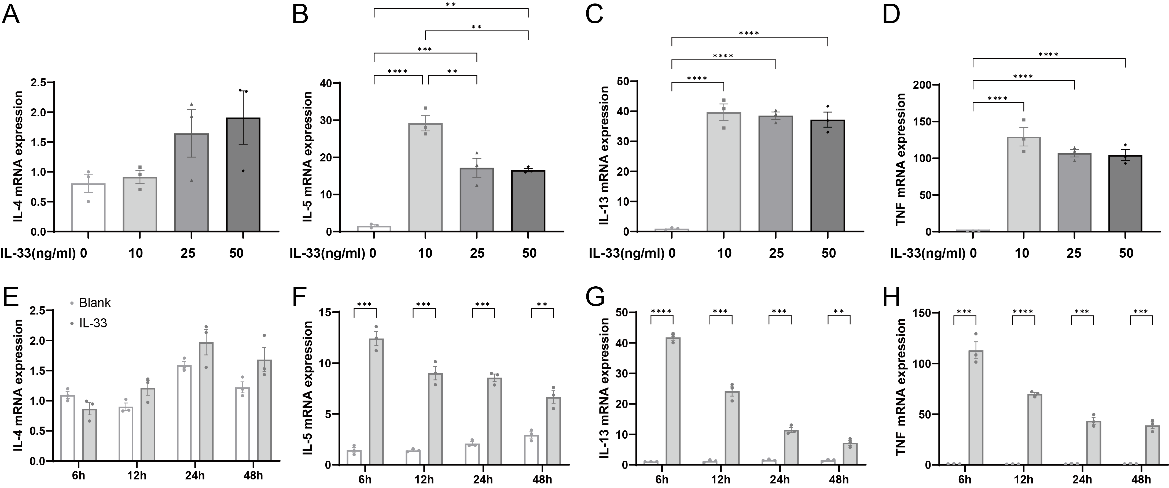
**Supplementary Figure 3**. Effect of IL-33 on the expression of IL-4, IL-5, IL-13 and TNF in mast cells. The mRNA expression levels of (A) IL-4, (B) IL-5, (C) IL-13 and (D) TNF were analyzed using qPCR assay in human mast cells treated with different concentrations of IL-33 (0/10/25/50 ng/ml) for 6 hours. The mRNA expression levels of (E) IL-4, (F) IL-5, (G) IL-13 and (H) TNF were analyzed using qPCR assay in human mast cells treated with 10 ng/ml of IL-33 over a time course ranging from 0 to 48 hours. Unpaired t-test was used for comparison between the two groups. Asterisks indicate statistical significance, **P<0.01, ***P<0.001.


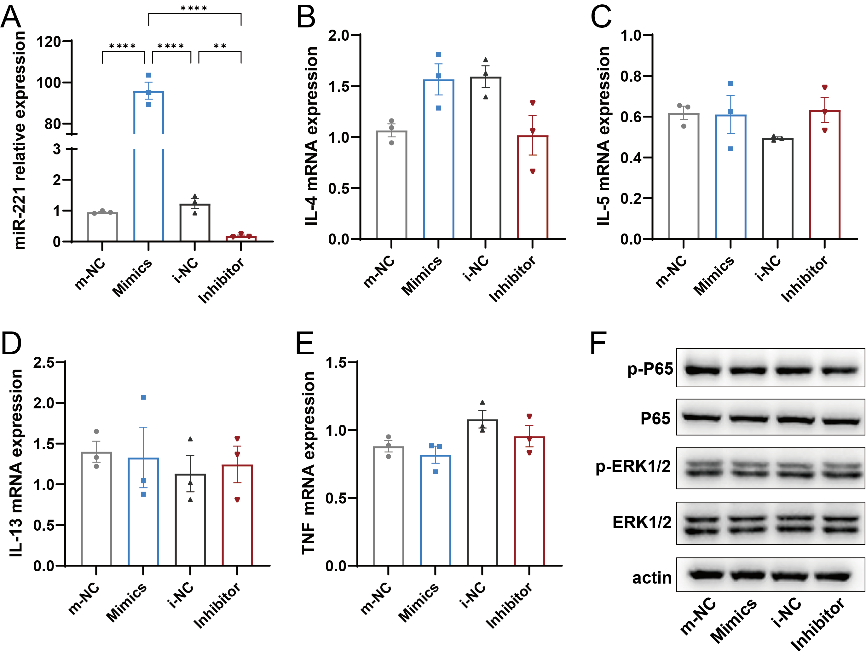
**Supplementary Figure 4**. Effect of miR-221-3p on cytokine expression and signal activation in mast cells. The expression of (A) miR-221-3p, (B) IL-4, (C) IL-5, (D) IL-13 and (E) TNF was analyzed using qPCR assay in mast cells transfected with miR-221-3p oligo for 24 hours. One-way ANOVA was used for comparison among the four groups. (F) The activation of P65 and ERK was determined by WB assay in mast cells under the same condition. The representative result of WB from 3 independent experiments is shown.


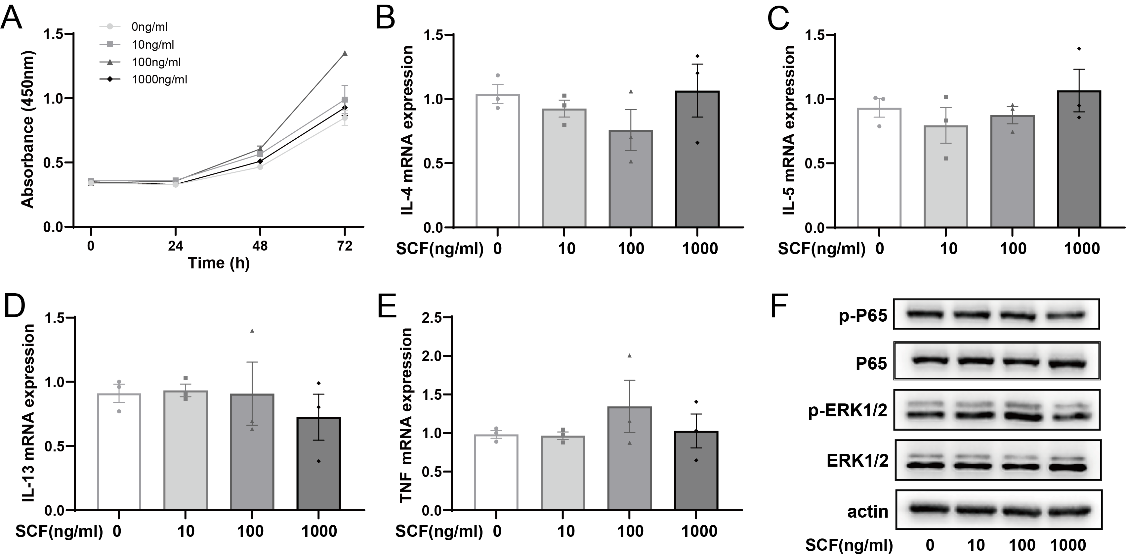
**Supplementary Figure 5**. Effect of SCF on the proliferation, signal activation and cytokine expression of mast cells. (A) The proliferation level of mast cells treated with different concentrations of SCF (0/10/100/1000 ng/ml) was assessed using CCK-8 assay at 24, 48 and 72 hours. The mRNA expression levels of (B) IL-4, (C) IL-5, (D) IL-13 and (E) TNF were analyzed using qPCR assay in mast cells treated with different concentrations of SCF (0/10/100/1000 ng/ml) for 6 hours. One-way ANOVA was used for comparison among the four groups. (F) The activation of P65 and ERK was determined by WB assay in mast cells treated with SCF (0/10/100/1000 ng/ml) for 6 hours. The representative result of WB from 3 independent experiments is shown.


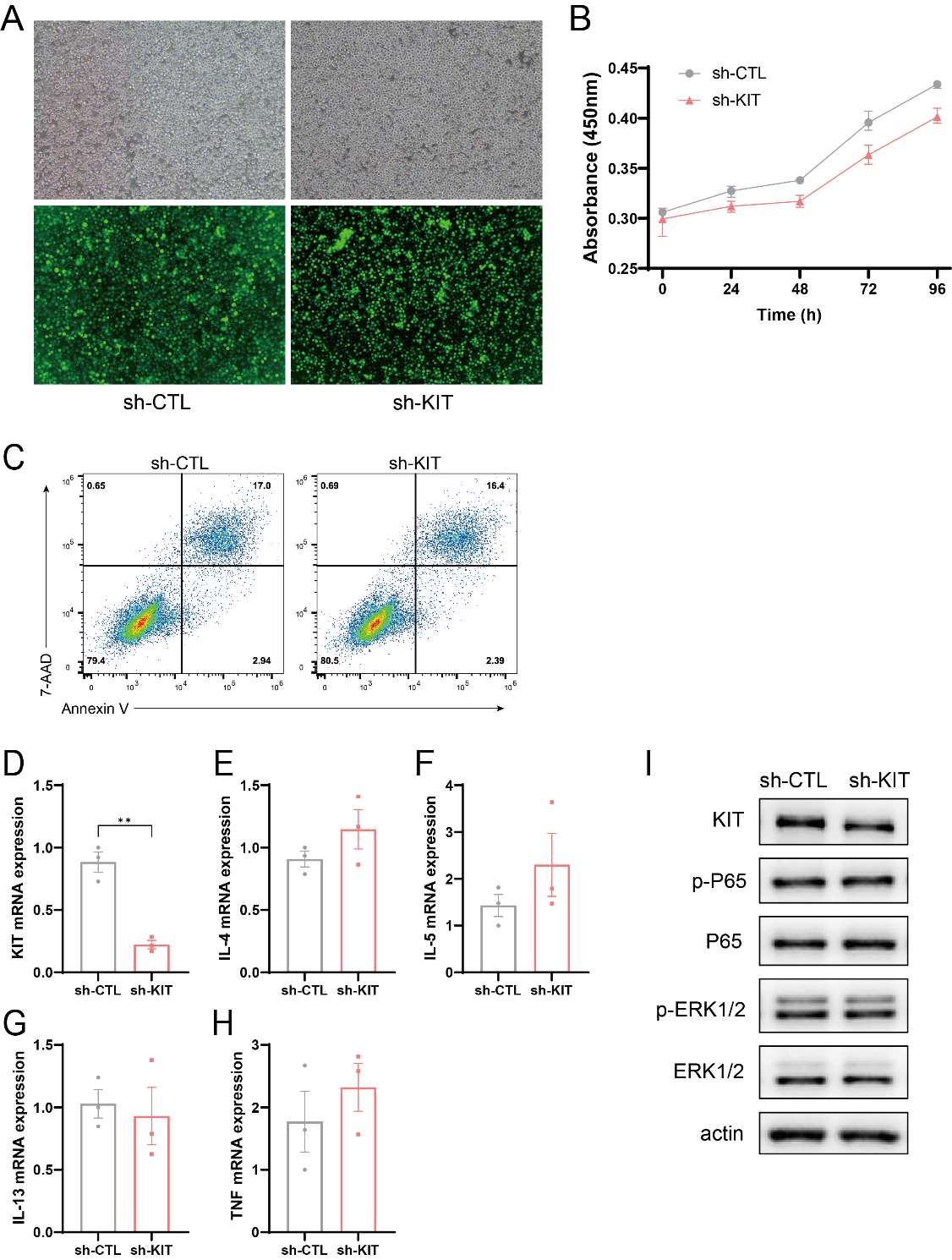
**Supplementary Figure 6**. Construction of robust lentiviral system for KIT knock-down in mast cells. (A) IF images demonstrating the eGFP (green) expression level of KIT knock-down mast cells (sh-KIT) and control mast cells (sh-CTL), the corresponding phase contrast images are also presented. (B) The proliferation level of sh-KIT mast cells and sh-CTL mast cells were assessed using CCK-8 assay at 24, 48, 72 and 96 hours. (C) The apoptosis level of sh-KIT mast cells and sh-CTL mast cells were assessed using flow cytometer assay. The mRNA expression levels of (D) KIT, (E) IL-4, (F) IL-5, (G) IL-13 and (H) TNF were analyzed using qPCR assay in sh-KIT mast cells and sh-CTL mast cells. Unpaired t-test was used for comparison between the two groups. (I) The protein expression of KIT and activation of P65 and ERK was determined by WB assay in sh-KIT mast cells and sh-CTL mast cells.
